# Simvastatin versus Calcium Hydroxide Direct Pulp Capping of Human Primary Molars: A Randomized Clinical Trial

**DOI:** 10.5681/joddd.2013.002

**Published:** 2013-02-21

**Authors:** Naser Asl Aminabadi, Ensiyeh Maljaei, Leila Erfanparast, Amir Ala Aghbali, Hamed Hamishehkar, Ebrahim Najafpour

**Affiliations:** ^1^Dntal and Periodontal Research Center, Tabriz University of Medical Sciences, Tabriz, Iran; ^2^Professor, Department of Pediatric Dentistry, Faculty of Dentistry, Tabriz University of Medical Sciences, Tabriz, Iran; ^3^Post-graduate Student, Department of Pediatric Dentistry, Faculty of Dentistry, Tabriz University of Medical Sciences, Tabriz, Iran; ^4^Assistant Professor, Department of Pediatric Dentistry, Faculty of Dentistry, Tabriz University of Medical Sciences, Tabriz, Iran; ^5^Associat Professor, Department of Oral Pathology, Faculty of Dentistry, Tabriz University of Medical Sciences, Tabriz, Iran; ^6^Drug Applied Research Center, Tabriz University of Medical Sciences, Tabriz, Iran

**Keywords:** Calcium hydroxide, direct pulp capping, simvastatin, hard tissue formation, inflammation

## Abstract

**Background and aims:**

The aim of present study was to investigate pulp-dentin complex reactions following direct pulp capping (DPC) with calcium hydroxide [Ca(OH)_2_] and simvastatin as pulp-capping materials in the primary human molars.

**Materials and methods:**

120 primary molar teeth which had to be extracted for orthodontic reasons were randomly allocated into four groups. Group Ι as a control, underwent DPC with calcium hydroxide. The dental pulp in group ІІ, ІІІ and ІV were directly capped with simvastatin-based materials at concentrations of 1, 5 and 10 µM, respectively. All of the teeth were restored with stainless steel crown. After a mean period of 7.41 months teeth were extracted and processed for histological examination and categorized in terms of hard tissue formation and pulp inflammation.

**Results:**

Teeth in group I had statistically favorable outcomes in hard tissue formation and pulp inflammation than did the groups ІІ, ІІІ and ІV (P < 0.001). Considering three different concentrations of simvastatin, the result showed a dose dependent trend. Teeth in group ІV showed significantly lower rates of hard tissue formation and higher rates of pulp inflammation and necrosis compared to those of groups ІІ (P < 0.05).

**Conclusion:**

The findings of this study depicted that healing with no inflammation and hard tissue formation following statin treatment occurs with a lower rate than that with the calcium hydroxide.

## Introduction

Direct pulp capping (DPC) involves the application of a medicament, dressing or dental material on the exposed pulp in an attempt to preserve its vitality.^1^ The rationale behind this treatment modality is the encouragement of the pulp to initiate a dentin bridge, thus “walling of” the exposure site.^2^ The success rate of this treatment is not particularly high in primary teeth and it therefore has limited application in this field.^2,3,4^ The high failure rate of DPC in primary teeth has been explained by undifferentiated mesenchymal stem cells in the primary pulp which may differentiate to odontoclasts, leading to the internal resorption.^1,2^

Many materials and drugs have been used as pulp capping agent to cover the exposure and initiate pulpal healing and/or hard structure repair. Calcium hydroxide [Ca(OH)_2_] has been the standard material by which all others were judged and has been generally accepted as the agent of choice.^5,6^ Nevertheless, calcium hydroxide has been shown to be soluble and degrade with time, and most dentine bridges beneath this agent, contain multiple tunnel defects, which often leads to failure of a long-term bacteriometic seal.^7,8^ However, the clinical success rate of calcium hydroxide is lower in primary teeth than in permanent teeth, possibly because of microleakage as well as induction of internal resorption.^9,10^ On the other hand, it has been noted that a high degree of success with DPC in primary teeth can be expected in carefully selected cases using specific criteria and treatment methods with proven materials.^11,12^

Therefore, the development of new pulp capping materials with a biologic ability to activate odontoblasts and accelerate dentin formation is desired. In an experimental setting, several growth factors were investigated and proved to induce odontogenic differentiation of dental pulp cells in vitro and to accelerate dentin formation in vivo.^11,13,14^ Among them, bone morphogenetic proteins were considered to be promising growth factors capable of promoting odontogenic differentiation.^15^ However, the possibility of unexpected side effects and the cost can be obstacles for their clinical application.

Statins are structural analogs of HMG-CoA (3-hydroxy-3-mthylglutaryl-coenzyme A). These drugs are the first-line for hyperlipidemia and it has been recognized to be a safe and low-priced drug as a result of its worldwide longtime usage.^16,17^ Moreover, statin has multiple functions including anti-inflammation, induction of angiogenesis and improvement of the vascular endothelial cell function. Another interesting and important function of statin is its effect on bone formation.^15^ It has been reported that several statins such as simvastatin and lovastatin have anabolic effects on bone metabolism at in vitro and in vivo studies.^18^ They promote mineralization in non-mineralizing osteoblasts through induction of BMP-2 and osteocalcin.^19^ Furthermore, in vitro studies showed that statins promote osteoblastic differentiation in mouse osteoblastic cells.^20^

In the light of these reflections, the present investigation was designed to investigate pulp-dentin complex reaction in terms of hard tissue formation and inflammatory response to statin as pulp-capping materials in the primary molars in vivo.

## Material and Methods

Fifty-four children aged 7-9 year old were chosen for this randomized clinical trial from attendances to the Department of Pediatric Dentistry, Tabriz University of Medical Sciences. The selected teeth were primary molars and had to be extracted for orthodontic reasons. The patients were referrals from orthodontic section for the purpose of comprehensive treatment and monitoring of selected teeth for serial extraction. The selected teeth were candidates for extraction on the scheduled time frame. However, these teeth needed dental treatment due to deep caries. Cases were selected considering the following inclusion criteria:

1. Complete physical and mental health without any confounding medical history
2. No history of spontaneous pain in teeth
3. No pathologic tooth mobility
4. Normal gingival and periodontal condition, without signs of pathology such as redness and swelling of the vestibule, draining sinus tracts or sensitivity to palpation in the vestibule
5. Teeth deemed restorable with a stainless steel crown
6. Absence of furcal/periapical radiolucencies or any pathologic root resorption
7. No pathology of the succedaneous permanent teeth follicles

The study procedure, possible discomforts or risks, as well as possible benefits were explained completely to the parents/legal guardians, and an informed consent form was obtained and recorded. This study was approved by the Ethics Committee and Research Council of the Tabriz University of Medical Sciences.

### Study Procedure 

Primary teeth with deep caries were randomly allocated into either DPC with calcium hydroxide or DPC with simvastatin groups. After administration of local anesthesia, teeth were isolated with rubber dam. Enamel and peripheral caries were excavated using a high-speed water-cooled air motor and round and fissure diamond burs. Soft, mushy carious tissue was removed manually with an excavator. All remaining caries from the cavity margins and pulpal floor were removed with a steel round bur running at slow speed. To ensure standardization, a pinpoint pulp exposure was performed with a dental explorer. True pinpoint exposure was surrounded by sound dentin and showed no bleeding or purulent/serous exudates at the exposure site. Sterile physiological saline was delivered by a syringe and needle to wash away dentin debris.

Once homeostasis was achieved, in Group І, calcium hydroxide powder [Ca(OH)_2,_ (Merck, Darmstadt, Germany)] was mixed with distilled water into a paste and delivered to the exposure site using small endodontic amalgam carrier. The paste was gently packed over the pulp at the exposure site with an amalgam condenser and small pellets of cotton wool. Excess agent was meticulously removed from cavity walls with an excavator.

Simvastatin at concentrations of 1 µM, 5 µM and 10 µM were delivered to the exposure site in groups ІІ, ІІІ and ІV, respectively, as described for group I. This solution was used along with sodium carboxyl methylcellulose high viscous (NaCMC H.V., Sigma-Aldrich, Germany) as an inert powder as carrier, which also had tissue adhesion property. In all groups, teeth were sealed with a 2-mm thick zinc oxide-eugenol base (Zonalin, Kemdent, UK). A primary molar stainless steel crown (Ion 3M Dental Products^®^; Loughborough, Leicestershire, UK) was fitted and cemented in place using glass-ionomer cement (GIC^®^; Ketac, 3M-ESPE AG, Seefeld, Germany).

### Histological Observations

After 6-9 months as part of the orthodontic treatment, teeth were extracted and then specimens were fixed with 4% paraformaldehyde solution and decalcified in ethylenediamine tetra acetic acid (EDTA) disodium salt (pH 7.4) for 10 days. The specimens were embedded in paraffin and cut sagitally at 5 μm in thickness. The sections were stained with hematoxylin and eosin to evaluate the hard tissue formation at the surface of exposed pulp and the general cellular architecture.

Hard tissue evaluation was based on the integrity of reparative dentin formed at the pulp exposure site. Specimen was graded and categorized as follows: Complete bridge formation; incomplete bridge formation; and no bridge formation. Regarding inflammation, the extracted teeth were classified according to the criteria described by Fuks et al^21^ as follows: none or mild inflammation; moderate inflammation; and severe inflammation and necrosis.^22^

### Statistical Analysis

The data were analyzed with chi square test and the statistical significance was set to 0.05. All data were presented as the means and standard deviation (SD).

## Results

54 children (25 females, 29 males; mean, 8.1 ±0.98 years) were allocated to the study groups. Four patients were excluded from the study because they did not complete the trial period. A total of 104 teeth were included in the analyses.

### Hard Tissue Formation 

**Figure 1:**
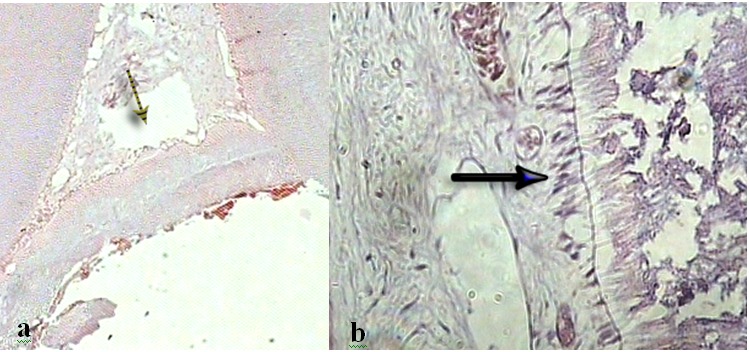
Histological feature demonstrating complete hard tissue formation in a tooth, treated by Ca(OH)_2_ (×4 magnification, a). A layer of odontoblasts in a teeth treated by Ca(OH)_2_ with complete hard tissue formation (×20 magnification, b).

A statistically significant difference was detected in complete bridge formation between group I and the other three simvastatin groups. Complete dentine bridge formation was seen in 55%, 20%, 7%, and 0% of treated teeth in groups I, II, III and IV, respectively (Figure 1). Overall, statistically significant differences was seen in hard tissue formation between calcium hydroxide group and the three simvastatin groups (X^2^ = 19.93, df = 3, P < 0.001; Tables 1 & 2).

**Table 1:** Number of specimens, from the cases which completed the study.

Material	HTFxxsup*xysup			Inflammation		Total
		No/mild	Moderate	Severe	Necrosis	
Calcium hydroxide	Complete	12	-	0	0	12
	Partial	2	-	2	0	4
	Non	0	-	0	6	6
	Total	14	-	2	6	22
Simvastatin 1	Complete	0	3	0	3	6
	Partial	6	0	0	0	6
	Non	6	0	3	9	18
	Total	12	3	3	12	30
Simvastatin 5	Complete	0	-	0	2	2
	Partial	2	-	0	0	2
	Non	2	-	8	14	24
	Total	4	-	8	16	28
Simvastatin 10	Partial	-	3	0	3	6
	Non	-	0	6	12	18
	Total	-	3	6	15	24

*HTF: Hard Tissue Formation

**Table 2:** Bridge state of specimens.

Material	HTF^*^	No HTF	Total
Calcium hydroxide	16	6	22
Simvastatin 1	12	18	30
Simvastatin 5	4	24	28
Simvastatin 10	6	18	24
Total	38	66	104

*HTF: Hard Tissue Formation

**Figure 2:**
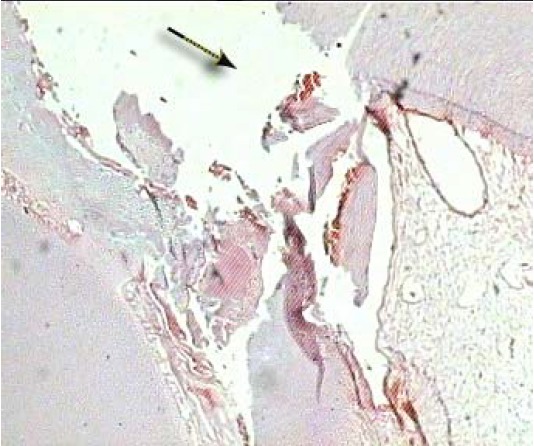
Histological feature, ×4 magnification. Arrow pointing incomplete hard tissue formation in a specimen treated by 1 µM simvastatin. A mild inflammation is detectable in the pulp.

There were also differences in bridge formation (partial and complete) between three different simvastatin concentrations (Figure 2). The highest value was detected in group ІІ with 40%, comparing to group ІІІ was statistically significant with 14.3% of cases (P = 0.029), and 25% (P = 0.245) in group ІV. The difference between group ІІ vs. ІV and group ІІІ vs. ІV did not reach to the statistical significant level (P < 0.05).

### Inflammation

Similarly a statistically significant difference was detected in inflammatory changes between group I and the other three simvastatin groups (X^2^ = 27.77, df = 6, P < 0.001). No inflammation or mild inflammation was detected in 63.6%, 40%, 14% and 0% of teeth in groups I, II, III and IV, respectively. The teeth in group І had significantly lower rate of inflammatory changes than did the other three groups (Tables 1 & 3).

**Table 3:** Pulp inflammatory state of the specimens.

Material		Inflammation		Total
	No./mild	Severe^*^	Necrosis	
Calcium hydroxide	14	2	6	22
Simvastatin 1	12	6	12	30
Simvastatin 5	4	8	16	28
Simvastatin 10	0	9	15	24
Total	30	25	49	104

*In this table to statistic reasons, we combined two groups of moderate and severe inflammation in to one group as severe inflammation group.

**Figure 3:**
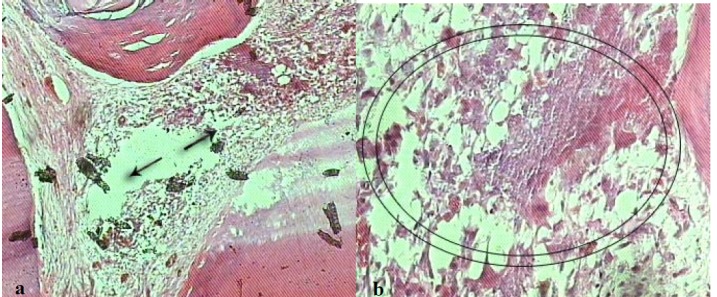
Histological feature. Demonstrating severe inflammation with areas of necrosis in a teeth treated by 5 μM simvastatin (×4 magnification, a); necrotic area in the same tooth (×20 magnification, b).

There were also differences in pulp necrosis between three different simvastatin concentrations (Figure 3). The highest number of pulp necrosis was detected in group IV with 62.5%, followed by 57.1% in group ІІІ and 40% in group ІІ. The difference between group ІІ and ІV was statistically significant (P = 0.002). The difference between group ІІ vs. ІІІ and group ІІІ vs. ІV did not reach to the statistical significant level (P < 0.05).

## Discussion 

The main objective of the present study was to investigate pulp-dentin complex reaction in terms of hard tissue formation and inflammatory responses following DPC with calcium hydroxide and simvastatin as pulp capping materials in human primary molars. To the best of our knowledge this study was the first attempt to apply simvastatin on human teeth as DPC material.

### Hard Tissue Formation

The results of present study revealed that hard tissue formation with calcium hydroxide was higher compared to that of different simvastatin concentrations. The high pH of calcium hydroxide (pH = 12.5) has an important role in the induction of hard tissue formation. An alkaline pH at the pulp surface creates sediment of calcium phosphate on the organic matrix, which induces the formation of a mineralized bridge over pulpal tissue.^23^

Lower success rate of simvastatin in our study may be attributed to the cytotoxic effect of statin that destroys dental pulp stem cells (DPSCs), odontoblasts or odontoblast-like cells, preventing exposure site to repair.^15^

We also compared the effects of three different simvastatin doses at 1 µM, 5 µM and 10 µM. Interestingly, our findings revealed a decrease in bridge formation by increasing the concentration of simvastatin. In agreement with this finding, in an in vitro and in vivo study conducted by Okamoto et al, all simvastatin concentrations (0.1, 1, and 10 µmol/L) showed hard tissue formation after 8 weeks, and DPSCs pretreated by simvastatin at 1 µmol/L formed a significantly larger amount of mineralized tissue in comparison to others simvastatin concentrations.^15^

The cell damage observed in the pulpal tissues treated with statin may be related to the effect of statin at high concentrations.^15^ Findings of the study conducted by Campos-Lara et al showed that fluvastatin has important activity against the three tumoral cell lines assayed by arresting the cellular growth in the G1-phase as well as significant decrease in the percentage of cells in the S phase. Moreover, cell growth was dose-dependent in the tumoral cell lines. Also a significant increase in the percentage of apoptotic cells for lovastatin, mevastatin and simvastatin were observed.^24,25^ It has also been suggested that statins inhibit the ras protein activation which is important in the regulation of cell differentiation and proliferation.^24^

The results of study conducted by Lin SK et al showed that simvastatin clearly inhibited the action of TNF-α in a dose-dependent pattern. Simvastatin attenuated bone resorption associated with apical periodontitis, possibly through suppressing the expression of Cysteine-rich 61 (Cyr61; a potential osteolytic mediator) in osteoblasts, and subsequently, macrophage chemotaxis into the lesions.^14^

In the present study, there were a number of teeth with no hard tissue formation, with all treatments, mostly belonging to group ІІІ and ІV. Although statins have been proved to enhance the promotion of odontoblastic differentiation,^26^ and the formation of mineralization nodules, simvastatin at high concentration decreased the ALP activity, as an index of odontoblastic differentiation in DPSCs, according to the simvastatin dose.^26^

### Inflammation 

Pulp inflammation and necrosis was higher in all studied simvastatin groups compared to calcium hydroxide group. Antibacterial, anti-inflammatory and tissue dissolving properties as well as osteogenic potential are among explanations for the high success rate of calcium hydroxide.^27,28,29,30^ Higher rate of Inflammation and pulp necrosis in simvastatin groups may be related to significant increase in the percentage of apoptotic cells due to cytotoxic effect of statins.^24,25^

Similarly, our findings revealed a higher rate of pulp inflammation and necrosis by increasing the concentration of simvastatin. These results may be mediated through this fact that statin in high concentration inhibits actin fiber formation and cell cycle progression, resulting in suppression of proliferation in DPSCs.^15^

**Figure 4:**
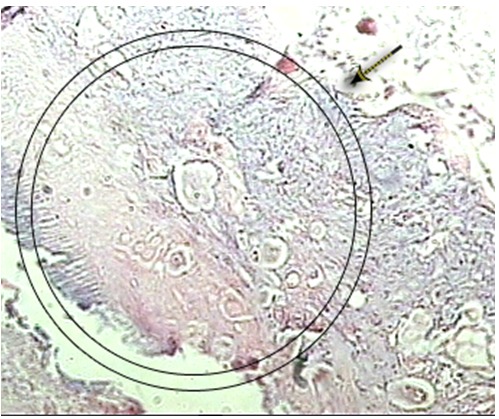
Histological feature, ×10 magnification. Osteocalcified band is observed inside the circle, while there is necrotic area detectable in some parts of the pulp in a tooth treated by 10 μM simvastatin.

An interesting finding in the present study was the occurrence of hard tissue formation along with a necrotic pulp in the same case (Figure 4). This finding may be attributed to the two-sided effect of simvastatin which has induced odontogenic gene expression in DPSCs and accelerated DPSC-mediated mineralized tissue formation, while suppressing the proliferation of DPSCs and its apoptosis effect.^15^

Further studies, including randomized controlled trials, are needed to examine the impact of statins on pulpal tissue along with antibacterial agents, considering its cytotoxic effect.

## Conclusions

The findings of this study depicted that healing with no inflammation and hard tissue formation following statin treatment occurs with a lower rate than that with the commonly used calcium hydroxide. Therefore, statins are not recommended as an alternative for calcium hydroxide as a DPC agent. Vital pulp therapy techniques such as DPC are challenging in pediatric dentistry. Development of new materials with a biologic ability to activate odontoblasts and accelerate dentin formation has become an issue of interest in recent years.
